# Experience of pediatric to adult transition in immunology services: patient experience questionnaire and micro-costing analysis

**DOI:** 10.3389/fimmu.2024.1270451

**Published:** 2024-03-05

**Authors:** Catherine King, Katie Ridge, James Smyth, Aisling M. Flinn, Timothy Ronan Leahy, Niall Conlon

**Affiliations:** ^1^ Diagnostic and Clinical Immunology, St. James’s Hospital, Dublin, Ireland; ^2^ School of Medicine, Trinity College Dublin, Dublin, Ireland; ^3^ Finance Department, St. James’s Hospital, Dublin, Ireland; ^4^ Department of Pediatric Immunology, Children’s Health Ireland at Crumlin, Dublin, Ireland

**Keywords:** inborn error of immunity, immunodeficiency, pediatric, transition, microcosting, patient experience survey

## Abstract

The effective transition from pediatric to adult care for individuals with chronic medical conditions should address the medical, psychosocial and educational needs of the cohort. The views and experiences of service users and their families are an integral component of service development. This study sought to evaluate the current provision of transition services from pediatric immunology services to adult immunology services for patients with a diagnosis of an inborn error of immunity at St. James’s Hospital, Dublin. We gathered patient perspectives on the experience of the transition process using a structured survey. In addition, we adopted a micro-costing technique to estimate the cost of implementing the current standard of care for these patients. Results of a micro-costing analysis suggest that the most significant component of cost in assessing these patients is on laboratory investigation, an area where there is likely significant duplication between pediatric and adult care. Perspectives from patients suggested that the transition period went well for the majority of the cohort and that they felt ready to move to adult services, but the transition was not without complications in areas such as self-advocacy and medication management. The transition process may benefit from enhanced communication and collaboration between pediatric and adult services.

## 1 Introduction

Transition is defined as the planned move of a patient’s care from a pediatric to an adult healthcare provider, accompanied by the graded transfer of responsibility for disease management from the parent to the young person themselves (1, 2). Concerted efforts have been made in the last two decades to better understand this often-turbulent time in the lives of young people with chronic diseases. Research into the factors that positively and negatively influence transition outcome has prompted the development of transition guidelines, however real-world application of these broad frameworks has been not without its difficulties. These difficulties have been particularly apparent in the setting of rare disorders where the number of experienced specialists is low and the access to guideline-recommended resources [such as psychological services, reproductive genetic counselling and allied health professionals (3)] can vary significantly between centers. The knock-on effect is that transitioning patients can be faced with long waiting times upon referral to the adult services and may seek to delay moving from the pediatric setting for fear of losing access to certain multidisciplinary supports (4).

Part of the difficulty in establishing a smooth transition process relates to the complexity of these patients’ underlying conditions. A significant number of combined immunodeficiencies, for example, may have non-immunological disease manifestations such as patients with 22q11.2 deletion syndrome who may require routine follow-up with ear, nose and throat specialists, cardiologists, endocrinologists, psychiatrists and orthopedic surgeons. Transition from the pediatric to the relevant adult service for each of these specialties may commence at different times and are frequently disjointed from one another. Furthermore, it is often the case that the pediatric hospital will have access to the majority of relevant subspecialties under one roof, but upon referral to adult services, care can become fragmented between different adult hospital sites based on appropriate local expertise and catchment areas. It is perhaps unsurprising that pediatric immunologists have reported greater challenges associated with transferring patients to the various non-immunology adult services for management of comorbidities, as opposed to the transition to the adult immunology service itself (5).

The heterogeneity within this cohort of transitioning patients cannot be overstated, with a huge variety of healthcare requirements represented in this group. A small proportion have minor immune system defects which may not require lifelong follow-up. Conversely, others have profound deficiencies which result in frequent hospitalization, significant burden of infection and requirements for multidisciplinary input. A “one-size-fits-all” approach to such a varied group is impractical. The significance of this period in a patient’s healthcare journey should not be underestimated however, with poor transitional care being linked with increased morbidity and mortality in a number of chronic diseases such as systemic lupus erythematosus (6), Type 1 diabetes (7), congenital heart disease (8), and sickle cell disease (9).

The downstream effects of a disordered transition between pediatric and adult services have been well-studied and can include a direct negative impact to the patient’s health as well as risks of the patient failing to adhere to treatment, disengaging from services and being lost to follow-up (10–12). In the setting of Inborn Errors of Immunity (IEI) this can include patients who underwent hematopoietic stem cell transplant at an early age and who warrant long-term follow up, patients with diagnoses which predispose them to malignancy who require surveillance monitoring, and patients taking a variety of immunosuppressing and immunomodulating treatments who need ongoing supervision from the adult immunology service.

We sought to assess current practices in the care of young adults in a large teaching hospital using a two-pronged approach. Firstly, we sought patient opinions on services in their current format in order to identify departmental strengths and unmet needs in this cohort. Given constraints in resources, the process of transition is required to be as lean and efficient as possible. To evaluate the cost of provision of current services, we then performed a micro-costing analysis to identify areas for improvement from a healthcare provider perspective.

## 2 Methods

Using hospital records, we identified all patients referred from the pediatric immunology service (in Crumlin Hospital, Dublin) to adult immunology services (in St. James’s Hospital, Dublin) between the years of 2015-2022. Inclusion criteria for the study were every patient referred from pediatric services to adult services with a (confirmed or suspected) IEI such as a diagnosis of antibody deficiency, combined immunodeficiency, defects in innate immunity, diseases of immune dysregulation and autoinflammatory syndromes. Also included were patients with hereditary angioedema and secondary hypogammaglobulinaemia. There were no specific exclusion criteria for the micro-costing analysis. For the questionnaire, patients were excluded if they were no longer routinely attending the adult service or could not be reached via telephone on three occasions. The study had full ethical approval by the Joint Research Ethics Committee at Tallaght University Hospital and St. James’s Hospital (Project ID 3575 and 3722 for the questionnaire and the micro-costing study respectively).

### 2.1 Patient experience questionnaire

Individuals identified via the above review of patient records were contacted to establish interest in partaking in a survey, and if in agreement, were emailed a patient information leaflet and a link to the online questionnaire. The survey could be completed by the patient or a parent (if the patient was unable to complete it themselves), however patient response was highlighted as being preferable where possible. The questionnaire was hosted on the online platform Qualtrics and responses were pseudonymized. The questionnaire was available to patients over a two-week period from 25^th^ May 2023 until 8^th^ June 2023. Questions were partly based on two previous surveys used in adolescents with chronic granulomatous disease and HIV (13, 14). Twenty-eight questions comprised the questionnaire which included a combination of close-ended questions, Likert scale ratings and five open-ended questions (**Supplementary Material 1**). Areas covered included basic demographic details, details on inpatient and outpatient hospital attendances, attitudes pre- and post- the transition process, self-management, and strategies for improving transitional care. Results were reported as descriptive statistics with measures of central tendency (mean) and with the use of Fisher’s exact test to determine nonrandom associations between categorical variables.

### 2.2 Micro-costing analysis

We sought to estimate the cost of initial outpatient clinic visits for patients with IEI transitioning from pediatric to adult immunology care. The typical pathway of new transitioning patients attending our clinic is detailed as follows. Patients are typically reviewed by either an immunology specialist registrar or an immunology consultant. All patients are reviewed by an immunology clinical nurse manager with particular attention paid to the administration of immunoglobulin replacement therapy where relevant. A staff nurse acts as a clinic facilitator performing blood pressure assessments and point of care tests as required. Blood tests are completed by a phlebotomist. Clerical officers provide administration support for the clinic coordinating the receipt of referrals, consultant triage, appointment scheduling and the management of all correspondence with both the patient and primary care providers.

Investigations performed at outpatient clinics are arranged based on clinical assessment and to some extent, are influenced by the investigations which have been carried out in pediatric care to date. For example, a patient who has not had genetic testing under pediatric care may warrant genetic testing under adult services. Typically, patients with established IEI attending adult immunology clinics for the first time will undergo routine blood tests including full blood count and lymphocyte subsets, renal and liver profiles, as well as the assessment of immunoglobulin, immunoglobulin subclasses and complement. Microbiological investigation for blood borne viruses is also typically completed.

Immediately after each clinic, patients are discussed as part of a multidisciplinary team meeting which is attended by immunology consultants, immunology specialist registrars and immunology specialist nurses. The purpose of this meeting is to review clinical histories, biochemical investigations and radiological investigations as appropriate and to plan ongoing clinical care. Both doctors and nurses use an electronic dictation system to dictate letters and electronic notes for patients. The clinical team are also responsible for the referral of patients to other specialties where appropriate.

For the purposes of the micro-costing analysis, a clinician reviewed medical records of the first outpatient appointment for all patients who met the above criteria. A cost-analysis was performed to estimate the direct implementation costs of existing clinical practice performed as part of the adult immunology outpatient care pathway. In collaboration with other team members, the clerical and clinical time involved in triaging and clinically reviewing these patients was estimated. Cost analysis also considered the consumables associated with clinic visits. All laboratory and radiological investigations that patients underwent as part of their first appointment with adult services were itemized. The total cost associated with the cohort was then divided by the total number of patients to generate a per patient cost. Unit costs used in the analysis were identified from publicly available data sources including the Irish Health Service Executive (HSE) published salaries of clinicians/clerical staff. Salary costs were estimated on the basis of the relevant HSE salary scales and following Irish health economic guidance (15, 16). The relevant staff positions considered for the purposes of salary scales were a Consultant Type A, Specialist Registrar (non-consultant hospital doctor), Clinical Nurse Manager II, Staff Nurse, Clerical Officer Grade IV and phlebotomist (16). We liaised with the hospital finance department, chief medical scientists and business managers in St. James’s Hospital.

## 3 Results

### 3.1 Patient experience questionnaire

Thirty-two patients were invited to participate in the study, of which 25 completed the questionnaire (78% response rate). Invited participants had a mean age of 18 years at the time of their initial attendance at the adult clinic, with a range from 16 years 4 months to 20 years 3 months. Demographic details of survey respondents are listed in **Table 1**. The majority of respondents were male (64%), with a mean current age of 21 and on average each patient was attending 3 other hospital specialties other than clinical immunology (ranging from 0 to 8). The highest level of education attained by the majority of patients (68%) was second level (high school), but included a variety of respondents, some of whom had never attended mainstream school and some who were pursuing university degrees. Forty percent of respondents had a family member with the same condition.

**Table 1:** Baseline characteristics of patients who completed questionnaire (N=25).

Sex:	
- Male - Female	16 (64%) 9 (36%)
Mean age at time of questionnaire (range)	21 years (16-27 years)
Survey respondent	
- Patient - Parent	16 (64%) 9 (36%)
Highest level of education attained by patient	
- Supported educational environment - Primary education (aged 5 to 12 approximately) - Secondary education (high school, aged 12 to 18 approximately) - Tertiary education (university, institute of technology, higher education institutions etc.)	1 (4%) 2 (8%) 17 (68%) 5 (20%)
Family member with same condition	
- Yes - No	10 (40%) 15 (60%)
Number of other hospital specialists the patient is regularly attending (apart from Immunology)	
- 0 or 1 additional specialists - 2 or more additional specialists	40% (10) 60% (15) (Average 2.68, with range 0-8)
Previously required inpatient admission relating to immunology diagnosis	
- Yes - No	56% (14) 44% (11)

Responses regarding anxiety levels pre-attending the adult hospital were mixed. There was 40% agreement with the statement “I was anxious about attending an adult hospital”, 40% disagreement, and 20% neutral. The majority of respondents disagreed with the statement that “The change from Pediatric to Adult clinic was stressful” (64% disagreement), and agreed that change went well (88% agreement), with three quarters of patients stating they felt ready to transition (76% agreement). There was a trend towards the transition period being more stressful for patients who attend at least two other hospital specialists, and for those without any affected family members, but this did not reach statistical significance (fisher exact value 0.0613 in both cases, significant at p <0.05). Anxiety, readiness, and perceptions of the process being stressful did not significantly differ between patient or parent respondents, male or female patients, or based on level of education.

Regarding self-management, only 32% of patients reported that they make their own appointments, from a median age of 18 years. Just over half of patients (56%) stated they attended alone for at least part of their immunology clinic review. The majority of patients reported they were responsible for their medications (68%), however only 44% of respondents would regularly link in with their pharmacy for medications and 44% would liaise with the team for a repeat prescription when needed. Seventy-six percent of respondents agreed with the statement “I have forgotten to take my medications or my infusion on at least one occasion”, with an average of 3 days missed at maximum.

The most commonly cited source of information on their immunological condition came from parents/families (reported by 72% of respondents), above their immunology doctor (56%), immunology nurse (24%) or general practitioner (8%). Sixty percent of patients stated they had also educated themselves on their condition, with specific online resources and support groups mentioned by 20% of this group.

Having not experienced a joint immunology transition clinic to date, 58% of respondents stated they would be in support of a joint clinic in the pediatric hospital to meet the adult team prior to transition. Fifty-four percent of patients reported that a young adult clinic in the adult hospital would be beneficial. Respondents who were regularly attending two or more other hospital specialties were significantly more likely to support a joint clinic than those attending immunology alone or just one additional service (Fisher exact value 0.0324, significant at p<.05). Similarly, patients with no affected family members were significantly more likely to support a joint clinic, than those who had an affected family member (Fisher exact value 0.0351, significant at p<.05). Support for a joint clinic or young adult clinic did not significantly differ amongst patient or parent respondents, male or female patients, or based on level of education. The reasons respondents gave for supporting a joint clinic were for enabling continuity of care (46%, 6/13 respondents who specified a reason) and to facilitate development of the new doctor-patient relationship (54%, 7/13 respondents). Those who were in favor of a young adult clinic cited benefits of having additional dedicated clinic time for discussing transition-related issues (45%, 5/11 respondents who specified a reason), and having the reassurance of other young patients attending the service (45%, 5/11 respondents). Those who did not support the notion of a joint clinic or young adult clinic generally reported that they did not feel it was necessary, that they were already familiar with the adult hospital, and that the current process worked well.

Open-ended questions regarding challenges, missing resources and suggestions for improvement ended the survey. Thematic analysis of respondents’ answers is detailed in **Table 2**. Female patients were significantly more likely to cite their biggest challenges as those involving self-advocacy and needing to form new interpersonal relationships with the adult team, whereas male patients were more likely to report the challenges of the physical logistics of changing hospital site and specific issues relating to their medical management (Fisher exact value 0.0034, significant at p <.05).

**Table 2:** Themes emerging from open-ended qualitative questions.

Common themes observed	Patient response (n, %)	Representative quotes
Challenges encountered during transition (n=22 responses, n=3 no comment)		
Physical logistics of a new hospital site	7 (32%)	“Learning my way around the hospital” “Lack of parking”
Self-advocacy	5 (23%)	“Having to become more independent” “Having to go on my own without my mam”
Development of a new doctor-patient relationship	3 (14%)	“New team. Was used to the same doctors and nurses in Children’s Hospital”
Changes to medical management	3 (14%)	“New medicine I was prescribed”
No challenges encountered	4 (18%*)	“We had no issues”
Suggestions for improvement (n=16 responses, n=9 no comment)		
Creation of a joint/young adult clinic	6 (38%)	“We have been to Young Adult clinics under different condition and found it very beneficial”
Improved coordination of pediatric-adult care	6 (38%)	“A care coordinator would be a great help”
Increased flexibility for patients with intellectual disability or autism spectrum disorders	2 (13%)	“Quiet clinic time at start or end of day”
Increased discussion of social aspects of adolescent care	1 (6%)	“Many young adults partake in illegal substances … should be discussed as openly and maturely as possible”
No improvements needed	1 (6%*)	“I was fully satisfied with my experience”
Resources lacking (n=23 responses, n=2 no comment)		
Pediatric service had been more directly accessible for all aspects of care	2 (9%)	“Results of all blood tests within days of clinic”
Lack of psychological support	2 (9%)	“Access to psychological support”
Coordinated care in one location	2 (9%)	“Paeds was all within one hospital, adult services are different clinics in different hospitals”
Reduced allowances for patients with intellectual disability or autism spectrum disorders	1 (4%)	“Waiting area in the Children’s Hospital is much quieter, which is better for my son”
No particular resources specified	16 (69%*)	“No, felt whole transition was excellent and nurses/doctors very understanding of young adult’s fears/anxiety”

*Percentages may not total 100 due to rounding.

### 3.2 Micro-costing analysis

Baseline characteristics of patients are listed in **Table 3**. In brief, data on forty-two patients were reviewed (24M:18F). Using a micro-costing approach, we established that a typical first visit to the adult immunology outpatient clinic between 2015-2022 for patients with a diagnosis of an IEI cost €416.69 per patient. The cost of the administration involved in patient referral, triage and scheduling was €23.88 per patient. The cost of clinical review by both a doctor and a nurse as well as clinical discussion at a multidisciplinary meeting amounted to €140.94 per patient. Laboratory and radiological investigations cost €243.84 per patient. The cost of consumables related to patient attendance was calculated at €8.03 per patient.

**Table 3:** Baseline characteristics of patients who underwent micro-costing analysis (N = 42).

Sex	Male: 24 (57%) Female: 18 (43%)
Mean age at first review (range)	18 years (16 years 4 months – 20 years 10 months
Mean wait time between receipt of referral and clinic appointment	216 days
Diagnosis	
**Antibody deficiency** CVID or evolving CVID XLA Symptomatic Selective IgA deficiency/IgG subclass deficiency/Specific Ab deficiency Secondary hypogammaglobulinaemia	13 patients (31%) 7 2 2 2
**Combined immunodeficiency** i) with syndromic features e.g. Trisomy 21, immune osseous dysplasia, NBAS deficiency, Kabuki Syndrome ii) without syndromic features (e.g. CARD-11 mutation) iii) 22q11 mutation iv) Hyper IgE syndrome (STAT3 loss of function)	14 patients (33%) 6 2 5 1
**Defects in innate immunity** i) Chronic granulomatous disease ii) WAS mutation iii) Complement deficiency iv) Chronic mucocutaneous candidiasis linked to STAT 1 gain of function mutation	5 patients (12%) 1 2 1 1
**Diseases of immune dysregulation** APECED EBV-driven HLH (without identified genetic defect in perforin, XIAP or SAP)	3 patients (7%) 2 1
**Other** Hereditary angioedema due to C1 inhibitor deficiency PTSPIP1-Associated Myeloid-related proteinemia Inflammatory syndrome (PAMI) Complex AI syndrome not otherwise defined Recurrent infections and bronchiectasis without identified immunological defect to date	7 patients (17%) 3 1 1 2
Treatment at time of referral (several patients prescribed more than one treatment modality)	
Immunoglobulin replacement Antimicrobial prophylaxis - Antibacterial prophylaxis - Antiviral prophylaxis - Antifungal prophylaxis Immunosuppression Granulocyte-Colony Stimulating Factor Previous bone marrow transplant for IEI Not taking any treatment for their underlying immunological diagnosis	12 (31%) 15 (36%) 3 (7%) 2 (5%) 4 (10%) 2 (5%) 1 (2%) 9 (21%)

## 4 Discussion

Available guidelines have highlighted several key areas that should be addressed in order to maximize success in transition (1–3, 17). Common themes include early commencement of the transition process (on an individual basis, considering patient maturity and psychological status), effective communication and cooperation between services, allowing the patient to develop autonomy in healthcare decision making, and involving family and carers through the process. Translating these detailed and notional guidelines into reality can be a challenge. The development of a specialty-specific transition policy which is at once all-encompassing (detailing the entirety of the biopsychosocial status of the patient, as well as previous treatments, investigations and comorbidities), yet innately flexible (allowing for each patient’s individual challenges) is a considerable task. Gaining direct feedback from the group at the center of this dynamic process provides a valuable insight into how we can better serve them going forwards.

Access to appropriate multidisciplinary input in adult immunology services varies significantly between countries and individual centers. Psychological support is cited in many of these guidelines as being a key component of transitional care but its widespread availability in the setting of IEI is lacking, even for patients who have undergone hematopoietic stem cell transplant for their underlying condition (18). For questionnaire respondents who felt that the adult immunology services were lacking certain essential resources, 29% of those related to psychological supports (2/7 respondents). This is of particular relevance when we consider that quality of life scores in adolescents with IEI who receive immunoglobulin replacement therapy are strikingly lower than scores in patients with diabetes, and comparable to those with cancer (19). With regards to multi-specialty involvement, survey respondents reported attending up to 8 other medical clinics apart from clinical immunology. It is often the case in the setting of a complex diagnosis which involves immunodeficiency, that the responsibility falls to the immunologist to coordinate care with the other involved specialists. This can come at the expense of patients forging a meaningful clinical relationship with their general practitioner. Primary care services are often overlooked during transitional healthcare planning. Our findings highlight the potential complexity facing primary care physicians who oversee the care of young adults with immunodeficiency transitioning across multiple specialties. It is imperative for patients, families and physicians that primary care doctors are integrated into transition plans and active issues (20).

Encouraging parental support while promoting increased self-efficacy in transitioning patients is also a common feature of aforementioned guidelines. A significant proportion of the available literature on pediatric transition involves conditions such as IBD and asthma which do not necessarily have as strong a genetic component as IEI. Forty percent (10/25) of our questionnaire respondents had a family member affected by the same condition as them. Following on from this, our data has also shown that transitioning patients’ most frequent source of information on their condition is their parents or family (reported by 72% of respondents), above their immunology doctor (reported by 56% of respondents). This is not unexpected given young people are dependent on parental guidance and input during clinic appointments, but is certainly something which merits attention in the transition period. It emphasizes the importance of engaging the patient themselves during clinic appointments with regards to all aspects of their care. We gained a further insight into the parental influence on patient care when we reviewed the nature of study respondents. The number of parental respondents to our questionnaire (n=9, 36%) was higher than anticipated, and higher than would account for the number of patients with intellectual disability who were invited to the study. This was likely due to patients whose listed phone number on their medical records were still their parent’s contact details as opposed to their own, to whom we had initially made contact. Despite our request that the patient be the one to complete the online survey if possible, it is clear that there were a number of parents who felt obliged to respond on their child’s behalf regardless. We had anticipated that parental responses would be a surrogate marker for patients with neurodevelopmental delay, but that was not the case based on the invited cohort and the number of parental respondents. This limited our ability to stratify questionnaire responses based on the presence of comorbid intellectual disability, which is a subgroup whose experience would have been particularly useful to know. It also strengthens the notion that self-advocacy and healthcare ownership continue to be challenging areas for some young adult patients beyond the initial transition period. For parents, the period of transition from pediatric to adult services requires them to reassess responsibility for their child’s management and to shift to a more secondary role in healthcare interactions, which some can find difficult (21).

Our results have also shown that patients with an affected family member are significantly less interested in attending a joint clinic prior to transition, compared to those without an affected family member. There are a number of reasons as to why this might be the case. Having witnessed an older sibling moving from the pediatric to adult setting, patients may already have a sense of familiarity with the adult hospital. They may also feel that the benefit of peer interaction at a transition clinic is less required if they already have a close contact with the same condition, unlike those without an affected family member who may value this more highly. The implications here are twofold. These findings highlight the undoubted need for targeting support and resources towards patients who do not have familial connections with the same condition, be it in the form of a longer appointment times, online resources providing information on their condition and links to patient support groups (all of which could be facilitated in a joint clinic). It also highlights that patients with affected family members may feel less of a need to engage fully with services in their own right and aligns with the earlier finding of parents being the prime source of medical information cited by respondents of our questionnaire. These findings exemplify the over-reliance that young adult patients may have on parental and familial experience even beyond the transition period. The requirement for patients to develop skills in self-advocacy is something which should be directly addressed by clinicians at clinic appointments during this time.

Building on this point, only 56% of patients in our survey stated they currently attended alone for any part of their clinic appointment, which is significantly lower than the rate of 72% reported by transitioning patients with CGD specifically (13). This may be influenced by the slightly older age of that study cohort (mean age of 23 years, versus 21 years in our group) and by the inclusion of patients with a comorbid intellectual disability in our cohort. A commonly encountered phenomenon in studies of transitioning young adults is that the patient may perceive the transition as having gone well, and may report high levels of readiness prior to that time, but these impressions are not always corroborated by measurable outcomes of successful transition. Over three-quarters of our survey respondents felt ready to transition, however less than one third currently made their own appointments with the clinic. Although 68% reported that they were responsible for their own medications, only 44% actively linked in with their pharmacy or the hospital for a repeat prescription. Similar outcomes have been observed in the setting of HIV and asthma, where patients who reported being ready for transition had stronger perceptions of medication independence, however this did not bear out in actuality with greater medication adherence or increased responsibility (22, 23).

Thematic analysis of qualitative survey responses revealed a distinction between male and female patients regarding the obstacles they encountered during the transition process. Female patients were significantly more likely to report difficulties relating to self-advocacy and the formation of new interpersonal relationships with the adult team during the transition process, and male patients were more likely to cite challenges with physical logistics of a new hospital and specific issues relating to their medical management. The emotional readjustment to new healthcare providers was reported as being one of the major barriers to a successful transition in a cohort of patients with systemic lupus erythematosus, of whom over 90% were female (6). This illustrates the importance of building a rapport with transitioning patients during initial visits with an adult team, and serves as a reminder that challenges perceived by one patient may be strikingly different to those encountered by another.

An often-repeated platitude in management strategy is “what cannot be measured cannot be improved”. While cost estimation, resource utilization and unit cost data are vital in all settings, it is of particular importance in the Irish setting currently as it prepares for the enhanced integration of pediatric and adult care through the co-location of a national children’s hospital and adult services (24). Micro-costing is a technique of cost estimation which gathers unit cost data to obtain precise estimates of economic costs. This “bottom-up” approach to costing healthcare is thought to be more accurate than a “top-down” approach, which involves a more general cost being applied to a particular healthcare service based on routinely available, per diem costs (25). Our data has suggested an initial clinic cost of €416.69 for new patients being transitioned to adult immunology services from the pediatric setting. Our analysis demonstrated that the cost of laboratory and radiological investigations in this cohort outweighed the cost of clinical assessment. In the absence of a formal transition protocol with no visibility of laboratory or radiological investigations across different hospital sites, there is undoubted replication at this initial clinic review which could be mitigated by enhanced coordination between centers. A recent survey of transitional practices carried out by the European Reference Network (ERN-RITA) reported that 79% of immunology services had full integration of patient records between the adult and pediatric healthcare providers, which is not the case in the Irish setting at present (5). Mitigation against the risks of a disjointed transition process could take the form of a joint clinic, an MDT discussion between pediatric and adult services, a nominated care coordinator, or a transition checklist whereby copies of all relevant serology, genetic reports and imaging results accompany each patient. Needless to say, a portion of previously completed investigations (genetic testing in particular) should be reconsidered over time and revisited with the identification of new monogenic disorders associated with IEI. Increased awareness of the costs associated with these patients provides real world data on a cohort which is often underestimated in hospital budgets and may be used to inform decision-makers in hospital management of unmet needs in this group. These results provide a snapshot of costs associated with a first visit from these patients to the adult service. The employment of a micro-costing approach to transition services for clinical immunology patients is novel. In order to better understand our results, we compared our data with a similar study completed by pediatric clinical immunology services assessing unit cost data for outpatient clinics evaluating drug allergy (26). The cost of outpatient clinic appointments in both studies was comparable (€518.36 in pediatric services versus €416.69 in adult services). Future work would benefit from a cost analysis of truly integrated pediatric and adult care, for example, through the costing of joint clinics or via a longitudinal costing study of healthcare interactions by these patients throughout their first year of adult clinics.

Our study has a number of limitations. Despite a reasonably good response rate of 78% our patient numbers are quite small (25 respondents). Patients were not surveyed at the same time point after transitioning from pediatric care, with some respondents having first been seen in the adult clinic 8 years prior to answering the questionnaire, versus others who had transitioned from pediatric services only 6 months prior to the study. This may contribute to a degree of recall bias in our older patient cohort. We did not directly ask survey respondents for their age at symptom onset, which would have been informative in establishing whether there was a significant difference in experience between patients diagnosed with IEI in infancy, versus those who presented in early adolescence. This study was completed in the adult hospital and we did not have access to pediatric costing data or medical records to evaluate indicators of patient readiness for transition (e.g. details on which patients may have attended their pediatric appointments by themselves, or taken responsibility for their own medications prior to transition), which would have been useful to contextualize our questionnaire results. Nor did we specifically ask questions regarding aspects of transition that went well upon transfer to adult services, which would have been instructive and likely encouraging. As above, we also had higher than anticipated numbers of parental respondents to the survey even in the absence of neurodevelopmental delay in their child. This highlights the limitations of using an online questionnaire platform for patient responses. Although we benefited from gaining broad insights into the cohort experience in a short space of time, there was less oversight available to us than would have been achieved via in-person interviews. Lastly, our study is limited by its lack of direct comparator for the micro-costing analysis but, as noted above, there is very little published data on costing in immunology clinical services to date.

In conclusion, this study has provided a useful analysis of the costs associated with the initial attendance of a patient with IEI transitioning to our service. This form of costing review could be reproduced by any other clinical specialty with the aim of rigorously evaluating departmental processes and as such, enabling service improvement. Unnecessary duplication of investigations can significantly contribute to the costs associated with new transitioning patients, which can be minimized by the implementation of formal transition protocols. Service improvement and generation of departmental protocols will only succeed however with the meaningful involvement of relevant stakeholders such as transitioning patients and their families. Several of our questionnaire findings highlight the complexity of balancing parental support and development of self-advocacy in young adult patients. Different patient populations can be faced with a variety of obstacles during this period (depending on gender and the presence or absence of a positive family history). We have gained valuable and relevant input from a cohort of young adults who have recently undergone this process, in particular perspectives on challenges and missing resources which will go on to inform our departmental protocols as we plan a formal transition policy.

## Data availability statement

The raw data supporting the conclusions of this article will be made available by the authors, without undue reservation.

## Ethics statement

The studies involving humans were approved by Joint Research Ethics Committee at Tallaght University Hospital and St. James’s Hospital. The studies were conducted in accordance with the local legislation and institutional requirements. The participants provided their written informed consent to participate in this study.

## Author contributions

CK: Conceptualization, Formal Analysis, Methodology, Writing – original draft. KR: Conceptualization, Formal Analysis, Methodology, Writing – original draft. JS: Resources, Writing – review & editing. AF: Conceptualization, Writing – review & editing. TL: Conceptualization, Writing – review & editing. NC: Conceptualization, Supervision, Writing – review & editing.

## Supplementary material

Supplementary Material 1 Patient experience questionnaire.

Supplementary Material 2 Patient information leaflet.
