# Supplementary material for: Experience of pediatric to adult transition in immunology services: patient experience questionnaire and micro-costing analysis
Source: Front Immunol. 2024 Mar 5;15:1270451. doi: 10.3389/fimmu.2024.1270451 (PMC10952820; doi:10.3389/fimmu.2024.1270451)
Supplement: Supplementary Material 1 — Patient experience questionnaire. [file DataSheet_1.docx]

EXperience of Pediatric to Adult Transition in Immunology services (EXPAT-Immunology)

**CONSENT**

1. I confirm that (please tick each option if in agreement)

- I have read the information sheet attached and understand my participation is voluntary.
- I agree to have my data processed in the method outlined in the email.
- I agree to partake in the study.

**DEMOGRAPHIC DATA**

1. Who is completing the study?

- I am (the patient)
- I am completing it on behalf of the patient. My relationship to the patient is: ______________________

**Please answer the following questions relating to the patient themselves.**

1. What is your current age?

______________________

1. What is your gender?

- Male
- Female
- Transgender
- Non-binary
- Prefer not to say

1. What is your occupational status? Choose as many as apply.

- In secondary school
- In college
- In a supported educational environment
- Working full time
- Working part time
- Not employed

1. What is the highest degree you have obtained?

- Primary school
- Junior Certificate
- Leaving certificate
- Level 6/Higher Certificate
- Level 7/Ordinary Bachelors degree
- Level 8/Honours Bachelors degree, or above

1. Do any of your family members have the same condition as you?

- Yes
- No

**HOSPITAL FOLLOW-UP**

1. Where was your **main place of residence** when you **first** attended the Adult Immunology Clinic in St James’s Hospital?

- In your family home (with your parents/guardians)
- In student accommodation
- Elsewhere (eg. With housemates / a partner / member of your extended family)

1. When was your **most recent appointment** with the Adult Immunology Clinic in St. James’s Hospital (approximately)?

- Within the last 6 months
- Within the last 12 months
- Within the last 18 months
- Over 18 months ago

1. Are you due to have any further appointments with the Paediatric Immunology team in Crumlin Hospital?

- Yes
- No
- Unsure

1. Are you still attending any **other Paediatric** medical services or clinics?

- Yes (please specify which below)

____________________________________________

- No

1. Approximately how many **other** medical specialists / clinics do you attend in total? *eg. cardiology, respiratory, endocrinology, ENT, gastroenterology, psychiatry etc*

______________________________________

1. Have you ever been an inpatient (stayed overnight) in hospital because of your Immunological condition?

- Yes
- No

**YOUR EXPERIENCE OF MOVING FROM PAEDIATRIC TO ADULT IMMUNOLOGY SERVICES**

1. Please indicate how strongly you agree or disagree with the following statements.

|  | Strongly agree | Somewhat agree | Neither agree or disagree | Somewhat disagree | Strongly disagree | Not applicable |
| --- | --- | --- | --- | --- | --- | --- |
| (i) I was anxious about attending an Adult Hospital |  |  |  |  |  |  |
| (ii) I was anxious about coming into an Adult Dayward for infusions |  |  |  |  |  |  |
| (iii) The change from Paediatric to Adult clinics was stressful |  |  |  |  |  |  |
| (iv) The change from Paediatric to Adult clinics went well |  |  |  |  |  |  |
| (v) I felt ready to transition to the Adult clinic |  |  |  |  |  |  |

**SELF-MANAGEMENT and MEDICATIONS**

Please answer True or False to the following statements.

|  | True | False | Not applicable |
| --- | --- | --- | --- |
| 1. I make my own clinic appointments  - If true, from what age? _______________ |  |  |  |
| 1. I have attended the Immunology Doctor on my own (for a part of, or for the entire clinic appointment)  - If true, from what age? _______________ |  |  |  |
| 1. I arrange my own transport to the hospital for my clinic appointments.  - If true, from what age? _______________ |  |  |  |
| 1. I am responsible for my medications. |  |  |  |
| 1. I link in with the pharmacy for my prescription. |  |  |  |
| 1. It is myself who requests a repeat prescription from my Immunology team or GP when my prescription is finished. |  |  |  |
| 1. I have forgotten to take my medications or my infusion on at least one occasion.  - If True, what is the longest period of time you have forgotten to take your medication/infusion? ie. a day, a week, a month   ____________________________ |  |  |  |

**SUPPORTS**

1. I have learnt about my condition and my treatment from *(select all that apply)*

- My parents or family
- My Immunology doctor
- My Immunology nurse
- My GP
- Other ________________­­­­­­­­­­­­­­­­­­________

1. I have educated myself about my condition

- True

If true, please specify (eg. particular websites, forums, books, etc). _____________________________

- False

**STRATEGIES FOR IMPROVING TRANSITIONAL CARE**

1. Do you think it would have been beneficial to meet a member of the Adult Immunology team during a **joint clinic with the Paediatric team** in Crumlin, before moving to St. James’s Hospital?

- Yes
- No

Please comment as to your choice:

____________________________________________________________________

____________________________________________________________________

____________________________________________________________________

____________________________________________________________________

1. Do you think it would be beneficial for the Immunology Department in St. James’s Hospital to have a specific **Young Adult Clinic**?

- Yes
- No

Please comment as to your choice:

____________________________________________________________________

____________________________________________________________________

____________________________________________________________________

____________________________________________________________________

1. Are there any particular **resources that you felt were lacking** from the Adult Immunology services, that you may have had access to in the Paediatric services?

*eg. Close links to other healthcare professionals, psychological support, social work, clinical nutrition, integrated care in one hospital, etc.*

Please comment as to how this affected you.

____________________________________________________________________

____________________________________________________________________

____________________________________________________________________

____________________________________________________________________

1. What were the **biggest challenges** you faced when moving from the Paediatric Immunology services to the Adult Immunology services?

____________________________________________________________________

____________________________________________________________________

____________________________________________________________________

____________________________________________________________________

1. Do you have any **suggestions** on how we can improve this process for our young adult patients in the future?

___________________________________________________________________

___________________________________________________________________

___________________________________________________________________

___________________________________________________________________

Thank you for your time in completing this questionnaire!
