# Supplementary material for: Experience of pediatric to adult transition in immunology services: patient experience questionnaire and micro-costing analysis
Source: Front Immunol. 2024 Mar 5;15:1270451. doi: 10.3389/fimmu.2024.1270451 (PMC10952820; doi:10.3389/fimmu.2024.1270451)
Supplement: Supplementary Material 2 — Patient information leaflet. [file DataSheet_2.docx]

| **PARTICIPANT INFORMATION LEAFLET** |
| --- |

| **EXperience of Paediatric to Adult Transition in immunology services (EXPAT): A patient experience questionnaire** |
| --- |

**Principal Investigators:**

Prof Niall Conlon^1^, Dr Catherine King^1^, Dr Katie Ridge^1^, Dr Ronan Leahy^2^, Dr Aisling Flinn^2^

1. Department of Clinical and Laboratory Immunology, St James’s Hospital, and

2. Department of Paediatric Immunology, Children’s Health Ireland, Crumlin.

Telephone: (01) 416 2928

Email: immunologymail@stjames.ie

We invite you to take part in a research study being carried out by the Immunology Department of St James’s Hospital. Before you decide whether or not you wish to complete the questionnaire, please read the information provided in this leaflet.

| **Why have I been asked to fill in this questionnaire?** |
| --- |

St James’s Hospital Immunology department receive referrals from many hospitals and GP practices. A small number of patients are referred to us from Paediatric Immunology services. These patients can often have a complex medical backgrounds, may be on specific treatments for their immune system, and often attend multiple other medical specialists.

The transition from Paediatric Immunology services to Adult Immunology services can be a big change for patients and we are interested in the patient experience of this process.

This questionnaire aims to understand how you felt about this transition.

The questionnaire can be filled out by yourself or a family member on your behalf, if you are unable to do so.

| **Do I have to take part? What happens if I say no?** |
| --- |

The questionnaire is voluntary and anonymous. We will not be able to identify you from your questionnaire responses.

Current or future medical care will not be affected by your decision to take part.

| **Are there any benefits to me or others if I take part in the study?** |
| --- |

The aim of this study is to help us understand the patient perspective on transitioning to Paediatric to Adult Immunology services.

What we learn could help us improve the experience for future patients going through this transition and identify particular areas of unmet need.

| **Will I be told the outcome of the study?** |
| --- |

The information collected will be analysed before results are available.

Results will be presented at medical meetings, conferences and in medical journals.

If you wish to know more about the study outcome, please enquire with the Immunology team directly.

| **PART 2 – DATA PROTECTION** |
| --- |

| **What information about me (personal data) will be used as part of this study? Will my medical records be accessed?** |
| --- |

The questionnaire is anonymous and we will not be able to identify you through your answers. Baseline data (sex and age) will be the only personal data recorded in the questionnaire.

The Qualtrics platform is GDPR (General Data Protection Regulation) compliant.

Your medical record will not be accessed for the purpose of this study.

| **PART 3 – COSTS, FUNDING & APPROVAL** |
| --- |

| **Will it cost me anything if I agree to take part?** |
| --- |

There is no cost to participate in the study.

The questionnaire will be made available to you via the online survey platform “Qualtrics”.

| **Will the results be used for commercial purposes?** |
| --- |

The data collected is intended to help and improve the care offered by the Immunology Department and is not for commercial purposes.

| **Has this study been approved by a research ethics committee?** |
| --- |

The Research and Innovation (R&I) Office in St James’s has given approval for this study to be carried out. Ethical approval has also been obtained from the TUH/JREC Joint Research Ethics Committee (JREC).

| **PART 4 – FUTURE RESEARCH** |
| --- |

| **Will my personal data be used in future studies?** |
| --- |

No.

| **PART 5 – FURTHER INFORMATION** |
| --- |

| **Where can I get information?** |
| --- |

**Principal Investigator:** Prof Niall Conlon, Consultant Immunologist.

**Data Controllers:** St James’s Hospital

**Data Processor(s):** Dr Catherine King, Dr Katie Ridge, Prof Niall Conlon.
